# EZH2 in Acral Lentiginous Melanoma: Molecular, Epigenetic, and Therapeutic Perspectives

**DOI:** 10.32604/or.2026.077913

**Published:** 2026-05-21

**Authors:** Daniel Arcuschin de Oliveira, Melissa Yoshimi Sakamoto Maeda Nisimoto, Jaciara Moreira Sodré Hunnicutt, Eduarda Massa Sartori, Amanda Fáris Marques, Francisco Macedo Paschoal, Luciana Cavalheiro Marti, Miriam Galvonas Jasiulionis, Miguel Sabino Neto, Renato Santos de Oliveira Filho

**Affiliations:** 1 Melanoma and Skin Tumors Sector, Plastic Surgery Discipline, Escola Paulista de Medicina, Universidade Federal de São Paulo EPM/UNIFESP, Rua Botucatu, 740, São Paulo, SP, Brazil; 2 Dermatology Discipline, FMABC University Center, Príncipe de Gales Street, 821, Santo André, SP, Brazil; 3 Department of Experimental Research, Hospital Israelita Albert Einstein, Rua Comendador Elias Jafet, 755, Morumbi, São Paulo, SP, Brazil; 4 Department of Pharmacology, Escola Paulista de Medicina, Universidade Federal de São Paulo EPM/UNIFESP, Rua Botucatu, 740, São Paulo, SP, Brazil

**Keywords:** Enhancer of Zeste Homolog (EZH)2, EZH2 inhibitors, acral lentiginous melanoma (ALM), trimethylation of histone H3 on lysine 27 (H3K27me3), therapy resistance, tazemetostat

## Abstract

Acral lentiginous melanoma (ALM) is characterized by a low mutational burden, frequent chromosomal rearrangements, and profound epigenetic dysregulation, distinguishing it from ultraviolet (UV)-induced melanoma. Among the epigenetic regulators, Enhancer of Zeste Homolog 2 (EZH2), the catalytic component of the Polycomb Repressive Complex 2 (PRC2), plays a central role in chromatin compaction and transcriptional repression through trimethylation of histone H3 on lysine 27 (H3K27me3). EZH2 overexpression or hyperactivation contributes to tumor progression, immune evasion, and therapeutic resistance. Recent multi-omic studies have highlighted the importance of EZH2 in regulating melanoma plasticity, immune modulation, and metabolic reprogramming. In ALM, where canonical oncogenic mutations such as BRAF V600E and NRAS Q61 are less frequent, EZH2-driven epigenetic mechanisms may play an even more dominant role in tumor initiation and progression. Pharmacological inhibitors of EZH2, including tazemetostat, have shown promise in preclinical melanoma models by restoring antigen presentation, enhancing CD8^+^ T-cell infiltration, and reversing transcriptional programs associated with immune resistance. This review aims to summarize the role of EZH2 in the molecular pathogenesis of ALM, emphasizing its contributions to epigenetic regulation, tumor plasticity, and immune escape, and discusses emerging therapeutic strategies targeting EZH2-mediated pathways to improve outcomes for this aggressive melanoma subtype.

## 1 Introduction

According to GLOBOCAN 2022, global cancer incidence reached approximately 20 million new cases worldwide, excluding non-melanoma skin cancers, underscoring the growing burden of malignant diseases across populations. Although cutaneous melanoma accounts for approximately 1.7% of all cancer diagnoses globally (331,647 cases/year), yet it is responsible for a disproportionate number of cancer-related mortalities due to its aggressive biological behavior, high metastatic capacity, and profound impact on patient survival [1,2]. Notably, therapeutic advances over the past decade have substantially reshaped the clinical landscape of metastatic melanoma, as evidenced by the approval of more than ten targeted or immunotherapy agents since 2011 [3,4].

Within the heterogeneous group of cutaneous melanomas, acral lentiginous melanoma (ALM) represents a distinct clinicopathological entity, accounting for approximately 2–8% of cases worldwide. This subtype arises predominantly in sun-protected sites such as the palms, soles, and nail apparatus and occurs more frequently in individuals with darker skin phototypes. Importantly, ALM constitutes a biologically distinct subtype by unique etiological, genetic, and molecular features when compared with other forms of cutaneous melanoma [5,6,7,8]. Histologically, ALM is defined by a lentiginous growth pattern and radial proliferation of melanocytes along the dermo-epidermal junction. Clinically, ALM is frequently diagnosed at more advanced stages owing to diagnostic challenges, which contribute to its comparatively poorer prognosis relative to other cutaneous melanoma subtypes [5].

Despite representing only 2–3% of melanomas in Western populations, ALM is the most prevalent melanoma subtype among individuals with Black skin, as well as Asian and Hispanic populations, and is associated with inferior prognosis. In contrast to UV-driven melanomas, ALM is primarily linked to genomic instability, including recurrent copy number alterations and mutations in genes such as *KIT*, *NF1*, *TERT*, and *TP53*. Its anatomical localization in less visible areas and frequent resemblance to benign lesions commonly delay diagnosis and adversely affect survival. Dermatoscopic findings, particularly the parallel ridge pattern, together with lentiginous histopathological features, are essential for accurate diagnosis [9]. Standard management includes wide local excision, with sentinel lymph node biopsy providing important prognostic and staging information. In advanced disease, immunotherapy remains the standard of care, although response rates are generally lower than those observed in non-acral melanoma subtypes. Furthermore, racial and socioeconomic disparities significantly influence clinical outcomes, highlighting the need for improved awareness, earlier detection, and tailored therapeutic strategies for ALM [10].

Given the aggressive behavior and distinct molecular landscape of ALM, epigenetic regulators have emerged as critical drivers of melanoma biology. In this context, Enhancer of Zeste Homolog 2 (EZH2) serves as the catalytic subunit of the Polycomb Repressive Complex 2 (PRC2), which mediates transcriptional repression through trimethylation of histone 3 (H3) at lysine 27 (H3K27me3). EZH2 plays a pivotal role in melanocyte differentiation, tumor suppression, and immune regulation, and is frequently hyperactivated in melanoma through gene amplification, overexpression, or aberrant upstream signaling. This dysregulation promotes transcriptional reprogramming, cellular dedifferentiation, and immune evasion, thereby contributing to melanoma pathogenesis, including ALM [11,12]. Consequently, pharmacological targeting of EZH2 has emerged as a promising therapeutic avenue for ALM, particularly in combination with immunotherapy-based approaches [13].

### Genomic Architecture and Clonal Evolution in Acral Melanoma

This section aims to summarize the current understanding of the genomic architecture of ALM, highlighting key driver alterations, mechanisms of clonal evolution, and their implications for tumor progression, immune escape, and therapeutic vulnerability.

Comprehensive genomic analyses of large melanoma cohorts, including more than 180 tumors analyzed by whole-genome and whole-exome sequencing (WGS/WES) and supported by validation cohorts, have revealed that acral melanoma exhibits a molecular landscape distinct from conventional cutaneous melanoma.

In contrast to UV-associated subtypes, acral melanoma displays a low ultraviolet (UV) mutational signature and is dominated by genomic instability, with widespread copy number alterations rather than UV-induced point mutations. Recurrently altered genes include *BRAF* (B-Raf proto-oncogene, serine/threonine kinase), *NRAS* (neuroblastoma RAS viral oncogene homolog), *KIT* (KIT proto-oncogene, receptor tyrosine kinase), *PTEN* (phosphatase and tensin homolog), and *TYRP1* (tyrosinase-related protein 1), together with frequent amplifications of *CCND1* (cyclin D1), *CDK4* (cyclin-dependent kinase 4), *MDM2* (MDM2 proto-oncogene, E3 ubiquitin protein ligase), and *TERT* (telomerase reverse transcriptase). This alteration converges on pathways regulating proliferative signaling, evasion of growth suppression, genomic instability, and replicative immortality, with prominent involvement of *MAPK* (mitogen-activated protein kinase), *PI3K*/*AKT* (phosphatidylinositol 3-kinase/protein kinase B), *WNT* (Wingless/Integrated signaling pathway), *NOTCH* (Notch signaling pathway), and cell-cycle control networks. Collectively, these findings indicate that acral melanoma is largely copy-number driven and harbors therapeutic vulnerabilities distinct from those of UV-associated melanomas, supporting the need for subtype-specific treatment strategies [14].

Genetic susceptibility to cutaneous melanoma involves both high-penetrance germline mutations and polygenic risk. Key predisposition genes include *CDKN2A* (cyclin-dependent kinase inhibitor 2A), *CDK4*, *BAP1* (BRCA1-associated protein 1), *MITF* (microphthalmia-associated transcription factor), *TERT*, as well as genes involved in telomere maintenance such as *POT1* (protection of telomeres 1), *ACD* (adrenocortical dysplasia protein homolog, also known as TPP1), *TERF2IP* (telomeric repeat-binding factor 2–interacting protein), and pigmentation-related genes including *MC1R* (melanocortin 1 receptor) [5]. Notably, several of these pathways, particularly those governing cell-cycle regulation and telomere integrity, are also recurrently disrupted at the somatic level in acral melanoma, highlighting a convergence between inherited susceptibility and tumor evolution.

In this line, acral melanoma frequently harbors alterations in key cell cycle regulators, most prominently amplifications of *CCND1* and *CDK4*, along with genetic and regulatory alterations affecting *TERT*. Accumulating evidence indicates that *TERT* promoter mutations and telomerase dysregulation promote replicative immortality, tumor progression, and clonal evolution in melanoma, with particular relevance in acral subtypes, where these mechanisms may partially compensate for the typically low mutational burden observed [7,15]. These genomic events interact with epigenetic mechanisms and transcriptional regulators, shaping intratumoral heterogeneity and influencing disease aggressiveness and therapeutic response [15].

Consistent with its non–UV-driven etiology, acral melanoma is characterized by a predominance of structural DNA alterations, including chromosomal amplifications, deletions, and rearrangements, resulting in an evolutionary trajectory less dependent on UV-induced point mutations [8]. Recurrent events include amplifications of *CCND1*, *CDK4*, and *TERT* [7,15], as well as alterations in components of the SWI/SNF chromatin remodeling complex, such as *ARID1A* and *ATRX*, and mutations in *NF1*, leading to constitutive activation of the MAPK pathway [16].

This genomic architecture promotes the emergence of heterogeneous tumor subclones with variable dependence on oncogenic pathways, including *MAPK*, *PI3K*/*AKT*, and *KIT*, as well as telomere dysfunction mechanisms that sustain uncontrolled proliferation [7,8,15]. Clonal heterogeneity not only fuels tumor progression and metastatic potential but also underlies intrinsic and acquired resistance to targeted therapies and immunotherapy. Distinct subclones may display variable immunogenic profiles promoting immune evasion through multiple mechanisms, including impaired MHC class I–mediated antigen presentation by tumor cells, dysfunctional antigen presentation by antigen-presenting cells, loss of neoantigen expression, and enhanced engagement of immune checkpoint pathways, such as PD-1/PD-L1 (programmed cell death protein 1/programmed death-ligand 1) signaling and enhanced CTLA-4–mediated T-cell inhibition. This dynamic interplay between genetic evolution and immune evasion shapes the tumor microenvironment (TME), favoring immune-resistant clones and limiting the durability of therapeutic responses.

#### Translational Implications of Genomic and Multi-Omics Profiling in Acral Melanoma

From a translational standpoint, the application of multigene panels, next-generation sequencing (NGS), and rare variant analysis has improved the identification of individuals at increased melanoma risk, and enables more detailed characterization of clonal complexity in ALM [8,16]. These advances support more precise genetic counseling and individualized surveillance strategies, and the future integration of genetic and clonal stratification into screening algorithms and therapeutic decision-making, reinforcing the importance of precision genomics in the management of acral melanoma [8,16].

Finally, integrated and spatial multi-omics analysis examining the progression of acral melanoma from *in situ* to the invasive stage, has shown that vertical invasion occurs early and follows a predominantly monoclonal trajectory lead by the acquisition of key oncogenic drivers, including *NRAS*, *KRAS*, *NF1*, or *KIT*. Three molecular subtypes were identified, with the most aggressive subtype characterized by high genomic instability and TME enriched in immunosuppressive APOE^+^/CD163^+^ macrophages. These cells promote tumor progression via the *IGF1–IGF1R* axis, as confirmed by spatial and functional analyses. Notably, combined APOE/CD163 expression emerged as a robust prognostic biomarker, providing a foundation for early risk stratification and the identification of novel therapeutic targets in acral melanoma [17].

Taken together, these findings establish acral melanoma as a biologically distinct, copy number–driven melanoma subtype in which inherited susceptibility, structural genomic instability, epigenetic and transcriptional reprogramming converge to drive early clonal evolution, tumor heterogeneity, and immune escape. The predominance of cell-cycle dysregulation, telomere maintenance alterations, and chromatin remodeling defects, coupled with a low UV mutational burden, defines a tumor evolutionary trajectory that differs fundamentally from that of UV-associated cutaneous melanoma. This unique genomic and microenvironmental context not only explains the aggressive clinical behavior and reduced responsiveness to immunotherapy observed in acral melanoma but also highlights specific vulnerabilities that may be exploited therapeutically (Table 1). Importantly, the integration of genomic, clonal, and spatial multi-omics data provides a framework for improved risk stratification, biomarker discovery, and the development of subtype-specific precision medicine strategies tailored to acral melanoma.

**Table 1:** Comparison between Acral Lentiginous Melanoma (AML) and Cutaneous Melanoma UV-associated.

Feature	Acral Lentiginous Melanoma	Cutaneous Melanoma (UV-Associated)
**Epidemiology**	Rare in Western populations (2–3%), most common subtype in individuals with Black, Asian, and Hispanic populations	Most common melanoma subtype in fair-skinned populations
**Anatomical location**	Palms, soles, nail apparatus (sun-protected sites)	Sun-exposed skin (trunk, limbs, head and neck)
**Primary etiological factor**	Not primarily ultraviolet (UV)-driven	Strongly associated with UV radiation
**UV mutational signature**	Low or absent	High
**Overall mutational burden**	Low	High
**Dominant genomic alterations**	Copy number alterations, chromosomal amplifications/deletions, structural rearrangements	UV-induced point mutations
**Key recurrent genomic events**	Amplifications of CCND1, CDK4, TERT, MDM2; alterations in KIT, NF1, ARID1A, ATRX. Low index of Mutations on NRAS (10–30%) and BRAF (21%)	Mutations in BRAF (V600E), NRAS, NF1
**Immune landscape**	Lower neoantigen load; immunosuppressive TME common	Higher neoantigen load
**Prognosis**	Generally worse	Mare favorable, especially with early detection

**Abbreviations:** CCND1 (cyclin D1); CDK4 (cyclin-dependent kinase 4); TERT (telomerase reverse transcriptase); MDM2 (MDM2 proto-oncogene, E3 ubiquitin protein ligase); KIT (KIT proto-oncogene, receptor tyrosine kinase); NF1 (neurofibromin 1); ARID1A (AT-rich interaction domain 1A); ATRX (alpha thalassemia/mental retardation syndrome X-linked); NRAS (NRAS proto-oncogene, GTPase); BRAF (B-Raf proto-oncogene, serine/threonine kinase); TME (tumor microenvironment).

## 2 Epigenetic Alterations in Acral Melanoma

This section discusses the major epigenetic mechanisms involved in ALM, including DNA methylation, histone modifications, and chromatin remodeling, and highlights how these processes contribute to tumor progression, immune evasion, and therapeutic resistance.

### 2.1 DNA Methylation Signatures and Prognostic Biomarkers in Acral Melanoma

Epigenetic regulation plays a central role in the pathobiology of ALM, particularly considering its low mutational burden and dependence on non-classical mechanisms of malignant transformation. Recurrent epigenetic alterations include promoter hypermethylation of tumor suppressor genes, microRNAs dysregulation, and the upregulation of histone methyltransferases such as EZH2 and G9a, which drive chromatin remodeling and impair cellular differentiation [18]. Among these, G9a has emerged as a promising epigenetic therapeutic target in melanoma, as it modulates critical pathways including Wnt, Notch, and autophagy. Notably, pharmacological inhibition of G9a has shown potential to enhance anti-tumor immune responses, particularly when combined with immune checkpoint blockade [19].

Beyond genetic alterations mentioned before, aberrant DNA methylation has emerged as a strong predictor of tumor aggressiveness and disease-specific survival in acral melanoma. Using the Illumina Infinium Methylation EPIC (850K) platform, distinct epigenetic signatures were identified among primary lentiginous acral melanoma (PALM), non-lentiginous acral melanoma (NALM), and metastatic acral melanoma (MALM). Notably, PALM and NALM displayed clearly divergent methylation profiles, with NALM associated with poorer clinical outcomes. Hypermethylation of *HHEX* and *NELFB* correlated with an increased risk of lymph node metastasis and unfavorable prognosis, whereas *IFITM1* and *SIK3* were strongly associated with metastatic progression. These epigenetic alterations also correlated with adverse clinicopathological features, including Breslow thickness, ulceration, mitotic activity, and perineural invasion. Collectively, these findings reinforce DNA methylation as a robust prognostic biomarker, a tool for risk stratification tool, and a potential therapeutic target in acral melanoma [20].

### 2.2 Histone Modifications and Chromatin Remodeling in Tumor Progression

Aberrant DNA methylation and histone modifications constitute major epigenetic mechanisms that regulate gene expression without altering the underlying DNA sequence, thereby critically influencing tumor progression, aggressiveness, and therapeutic resistance. Promoter hypermethylation of tumor suppressor genes, including *CDKN2A*, *PTEN*, and *RASSF1A*, together with global hypomethylation, contributes to genomic instability, tumor invasion, and metastatic potential. In this context, DNA methyltransferase (DNMT) inhibitors, such as azacitidine and decitabine, have shown promise in reactivating silenced tumor suppressor genes and enhancing tumor sensitivity to immunotherapeutic approaches.

In parallel, histone modifications, particularly those mediated by histone deacetylases (HDACs) and the methyltransferase EZH2, promote dedifferentiation, epithelial–mesenchymal transition (EMT), and immune evasion. Accordingly, pharmacological inhibition of HDAC, BET, and EZH2 inhibitors demonstrated antitumor activity and notable synergy with immune checkpoint blockade. Additionally, loss of TET2 function and global reduction of 5-hydroxymethylcytosine (5-hmC) have been identified as hallmarks of melanoma progression and immune resistance, further underscoring the central role of epigenetic dysregulation in disease evolution [20].

### 2.3 Mechanotransduction and Epigenetic Remodeling

A compelling example of the interplay between mechanical forces and epigenetic remodeling is the hypermethylation of the *PTEN* gene observed in acral arising in anatomical sites exposed to high mechanical stress, such as the heel, forefoot, and hallux. This epigenetic alteration is significantly associated with increased tumor thickness (higher Breslow index) and ulceration, reinforcing the connection between mechanical pressure, epigenetic dysregulation, and enhanced tumor aggressiveness [21].

In parallel, elevated levels of trimethyl-lysine (TML) have been detected in melanomas with increased metastatic potential, functioning as modulators of H3K9 and H3K27 methylation. These observations indicate that protein methylation metabolism is intrinsically linked to tumor plasticity and metastatic dissemination [1,9]. Within this framework, distinct methyltransferases exert complementary and cooperative functions: EZH2 primarily represses tumor suppressor and immune-related genes, whereas SUV39H1/2 and G9a, promote H3K9me3 deposition, facilitating heterochromatin formation and immune evasion [18,22]. Together, these mechanisms converge to establish a TME permissive to sustained growth, invasion, and therapeutic resistance [23].

### 2.4 Epigenetic Regulation of Tumor Immunity and Therapeutic Resistance

Importantly, in acral and mucosal melanomas, the low overall mutational burden suggests that epigenetic regulation may partially compensate for the limited availability of mutation-derived neoantigens by modulating antigen processing and presentation pathways, thereby influencing responsiveness to immunotherapy [24]. Consistent with this notion, experimental studies indicate that inhibition of EZH2 and other epigenetic regulators restores the expression of immune-related genes, increases lymphocytic infiltration, and improves the efficacy of immune checkpoint inhibitors [25]. Collectively, these findings position epigenetic dysregulation as a central determinant of ALM biology and highlight epigenetic targeting as a strategic therapeutic approach to overcome the intrinsic resistance observed in melanoma subtypes with low immunogenicity.

Accumulating evidence further indicates that resistance to therapy in advanced melanoma is associated with the establishment of an immunosuppressive phenotype characterized by diminished CD8^+^ T cell infiltration, reduced major histocompatibility complex (MHC) expression, and suppression of type I interferon signaling pathways, such as *cGas-STING* [26,27].

In this context, aberrant activation of the PRC2, driven by the methyltransferase EZH2, emerges as a key mechanism of non-genomic resistance, promoting epigenetic silencing of genes involved in both tumor suppression and antitumor immunity. EZH2 hyperactivation is detected in approximately 20% of melanomas associated with BRAF mutations, E2F activation, or noncanonical NF-κB signaling, resulting in coordinated repression of immune-relevant genes, including MHC molecules, chemokines, and interferon-response components. Beyond its tumor-intrinsic effects, EZH2 also modulates adaptive immunity, by sustaining the functional stability of regulatory T cells, thereby reinforcing tumor-associated immunosuppression. Together, these findings and observations provide a strong rationale for combining EZH2 inhibitors with immunotherapy or targeted therapy to reverse therapeutic resistance and restore melanoma immunogenicity [28,29].

### 2.5 Therapeutic Implications of Epigenetic Targeting in Acral Melanoma

**Figure 1:**
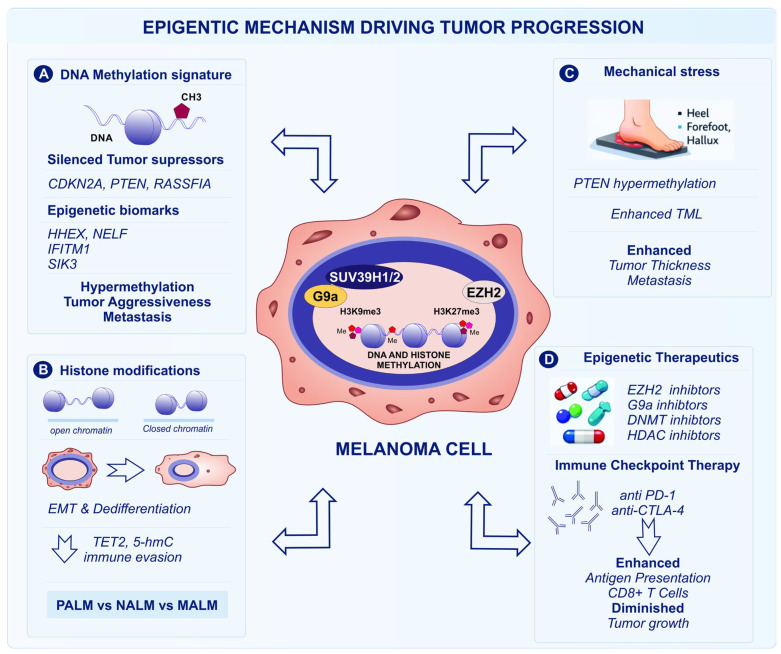
Epigenetic mechanisms driving tumor progression in acral lentiginous melanoma (ALM). This summarizes the major epigenetic processes involved in ALM. (**A**) DNA methylation mediated by DNA methyltransferases (DNMTs) leads to transcriptional silencing of tumor suppressor genes, including *CDKN2A* (*cyclin-dependent kinase inhibitor 2A*), *PTEN* (*phosphatase and tensin homolog*), and *RASSF1A* (*Ras association domain family member*); identifies prognostic methylation biomarkers such as *HHEX* (*hematopoietically expressed homeobox*), *NELF* (*negative elongation factor*); *IFITM1* (*interferon-induced transmembrane protein 1*), and *SIK3* (*salt-inducible kinase*), (**B**) Histone modifications mediated by methyltransferases such as *EZH2* (*enhancer of zeste homolog 2*) and *G9a* (*EHMT2*, *euchromatic histone lysine methyltransferase 2*) promote chromatin repression through H3K27me3 (Histone H3 lysine 27 trimethylation) and H3K9me3 (Histone H3 lysine 9 trimethylation) deposition, while loss of *TET2* (*ten–eleven translocation 2*) and reduced 5-hmC (5-hydroxymethylcytosine) contribute to melanoma dedifferentiation and immune evasion. (**C**) Mechanical stress in acral anatomical sites promotes epigenetic alterations, including PTEN hypermethylation and altered histone methylation dynamics, (**D**) Epigenetic therapies targeting DNMTs, EZH2, G9a, and HDACs may restore tumor immunogenicity and enhance responses to immunotherapy. Abb: PALM (Primary Acral Lentiginous Melanoma), NALM (Normal Acral Lentiginous Melanocyte), MALM (Metastatic Acral Lentiginous Melanoma), PD-1 (Programmed Cell Death Protein 1), CTLA-4 (Cytotoxic T-Lymphocyte–Associated Protein 4), CD (Cluster of Differentiation).

Taken together, these findings underscore that epigenetic dysregulation is not merely a secondary consequence of tumor evolution in ALM, but a central, driving force that compensates for its low mutational burden and shapes both tumor aggressiveness and immune escape. The convergence of DNA methylation changes, histone remodeling, and mechanotransduction-related epigenetic alterations highlights a non-genomic framework of disease progression that is particularly relevant to ALM and mucosal melanomas (Fig. 1). Importantly, the reversible nature of epigenetic modifications provides a unique therapeutic opportunity, positioning epigenetic regulators such as EZH2 and G9a not only as biomarkers of risk and resistance, but also as actionable targets to reprogram tumor–immune interactions. However, the clinical translation of epigenetic therapies will require careful integration with immunotherapy or targeted approaches, along with biomarker-driven patient stratification, to effectively overcome immune resistance and improve outcomes in these intrinsically low-immunogenic melanoma subtypes.

## 3 Biological Function and Relevance of EZH2

This section aims to summarize how EZH2 functions as a central epigenetic regulator of melanoma aggressiveness by sustaining stem-like states, enabling invasion and metastasis through H3K27me3-dependent programs, and promoting adaptive resistance to targeted and immune-based therapies. It also highlights mechanistic axes that control EZH2 abundance and recruitment, supporting EZH2 as a biomarker and as a combinatorial therapeutic vulnerability, including in the context of ALM.

### 3.1 EZH2 Sustains Melanoma Cancer Stem–Like Properties (MCSC)

The stem cell–associated survival protein EZH2 is highly enriched in melanoma cancer stem-like cells (MCSC). Genetic silencing of EZH2 or pharmacological inhibition using small-molecule inhibitors such as GSK126 or EPZ-6438, significantly reduces EZH2 activity [25], leading to impaired spheroid formation and diminished migratory and invasive capacities of MCSC. In parallel, the dietary cancer-preventive compound sulforaphane markedly suppresses MCSC survival, an effect mechanistically linked to EZH2 depletion. Notably, enforced EZH2 overexpression partially rescues sulforaphane-mediated inhibition of spheroid formation, migration, and invasion, supporting a causal role for EZH2 in maintaining stem-like properties. Collectively, these findings establish EZH2 as both a functional marker and a critical regulator of melanoma cancer stem–like cells, and suggest that sulforaphane limits melanoma tumorigenesis, at least, through a suppression of EZH2-dependent pathways [30].

### 3.2 EZH2-Linked Epigenetic Programs Integrate with SCNAs and Metastatic Signaling

In contrast, thick (>4 mm) cutaneous melanomas are characterized by a high burden of somatic copy number alterations (SCNAs), affecting major tumor suppressor genes, including *CDKN2A*, *PTEN*, *TP53*, as well as multiple oncogenes, and axonal guidance pathways, such as Slit/Robo, ephrins, and semaphorins, for the first time, suggesting a previously unrecognized contribution of neuronal signaling programs to melanoma aggressiveness. Additional alterations were detected in the *MAPK*, *NOTCH*, *HEDGEHOG*, and receptor tyrosine kinase signaling pathways [31]. Complementing these genomic observations, epigenetic regulation has emerged as a critical driver of melanoma invasion and metastasis. One study demonstrates that PGC1α expression in melanoma cells is epigenetically silenced through chromatin modifications involving H3K27 promoter trimethylation. Pharmacologic inhibition of EZH2 reduced H3K27me3 histone levels, restored PGC1α expression, and functionally suppressed invasive behavior in melanoma cells with silenced PGC1α. Consequently, inhibition of key components of this transcription–signaling axis, including TCF12, WNT5A, or YAP, blocked melanoma migration *in vitro* and metastasis *in vivo*. These findings indicate that the epigenetic control of melanoma metastasis involves modulation of PGC1α expression and its association with the tumor’s inherent metabolic state [32]. Consistent with this model, H3K27me3 is significantly associated with increased tumor thickness, lymph node involvement, and metastatic lesions, where it marks a dedifferentiated, invasive phenotype, supporting its potential use as a biomarker of aggressiveness and as a pharmacodynamic readout for EZH2-targeted therapies in clinical trials [33].

### 3.3 Epigenetic Plasticity Drives Resistance in BRAF-Mutant Melanoma through H3K27 Remodeling

Although *BRAF* mutations are not characteristic of ALM, they are highly prevalent in metastatic cutaneous melanoma and therefore provide an important framework for understanding how epigenetic mechanisms contribute to tumor progression and therapeutic resistance. Epigenetic plasticity is a central driver of therapeutic resistance in BRAF-mutant melanoma, as prolonged suppression of oncogenic *BRAF*, either through inducible loss of mutant BRAF or treatment with BRAF inhibitors such as vemurafenib, encorafenib, and dabrafenib, induces tumor regression followed by relapse driven by residual melanoma cell populations that undergo coordinated epigenetic reprogramming. This adaptive process involves conserved alterations in histone methyltransferase expression, including ASH2, EZH2, PRMT5, SUV39H1/2, and SMYD2, together with dynamic remodeling of histone marks characterized by reduced H3K9 and H3K27 methylation and increased H3K36 methylation, collectively promoting cell survival and drug tolerance [34]. Mechanistically, the BRAF^V600E mutation itself actively shapes the epigenetic landscape by inducing EZH2 expression via STAT3-dependent transcriptional activation, leading to PRC2-mediated H3K27me3 accumulation, silencing of tumor suppressor and melanocytic differentiation genes, and acquisition of a more aggressive, proliferative, and apoptosis-resistant phenotype. In parallel, BRAF V600E signaling governs an epigenetic–metabolic switch involving the transition from H3K27 methylation to acetylation (H3K27me3 → H3K27ac), whereby BRAF inhibition downregulates EZH2, enables KDM6A-mediated removal of H3K27me3, and activates the KDM6A–H3K27ac–BRD4 axis to upregulate *PGC1α*, thereby sustaining oxidative metabolism and facilitating survival under targeted therapy. Disruption of this methylation-to-acetylation switch impairs metabolic adaptation and sensitizes melanoma cells to BRAF^V600E inhibition, underscoring epigenetic remodeling of H3K27 as a critical link between oncogenic BRAF signaling, metabolic plasticity, and resistance, highlighting actionable therapeutic vulnerabilities within the PRC2–H3K27 pathway to enhance the durability of BRAF-targeted treatments [35,36].

### 3.4 Timing and Sequencing of EZH2 Inhibition with BRAF/MEK Blockade

Importantly, these findings raise key considerations regarding the timing and sequencing of EZH2 inhibition in combination with BRAF/MEK inhibitors. Early or adaptive co-targeting of EZH2 may prevent the establishment of drug-tolerant states by limiting chromatin reprogramming before metabolic and transcriptional escape programs become fixed [37]. Conversely, delayed EZH2 inhibition may be particularly effective in eradicating residual populations that emerge following prolonged MAPK pathway suppression, suggesting that both upfront and sequential combination strategies merit investigation [37].

### 3.5 EZH2 Inhibition as a “Cold Tumor” Conversion Strategy in Liver and Brain Metastases

Beyond targeted therapy resistance, the epigenetic framework defined by EZH2 also provides a compelling rationale for tumor with low T cells infiltration “cold tumor” conversion strategies, particularly in metastatic settings that are highly prevalent and clinically challenging in melanoma, such as liver and brain metastases. Both compartments are characterized by immunosuppressive microenvironments, as the liver is a tolerogenic immune circuit and the brain is constrained in immune surveillance, epigenetic reactivation of innate immune sensing may be especially important to unlock responsiveness to immune checkpoint blockade. Preclinical evidence showing that EZH2 inhibition restores STING expression, enhances type I interferon signaling, and increases CD8^+^ T-cell infiltration suggests that combining EZH2 inhibitors with STING agonists may be particularly effective in overcoming immune exclusion in these metastatic niches [38]. In the liver, where tolerogenic immune circuits dominate, and in the brain, where immune surveillance is intrinsically constrained, epigenetic reactivation of innate immune sensing may be a prerequisite for effective immunotherapy.

### 3.6 AP-2α_E2F_EZH2 Axis as a Metastasis-Specific Transcriptional Program and Therapeutic Entry Point

The transcription factor AP-2α (activating enhancer-binding protein 2 alpha), has emerged as a central regulator of metastasis in melanoma, overturning earlier views of this transcription factor as a mere tumor suppressor. Deletion of *TFAP2A* (transcription factor AP-2 alpha) abolishes metastatic competence without affecting tumor growth. Mechanistically, AP-2α acts as an epigenetic pioneer by sustaining E2F-axis gene expression through inhibition of the NuRD complex, thereby preserving H3 acetylation marks and maintaining key pro-metastatic and survival genes, including *EZH2*, *RAD51*, *RRM2*, and *E2F1/2/8*. Among the targets activated by AP-2α, EZH2 functions as a dominant epigenetic driver of aggressive melanoma sub-lineages. Pharmacologic EZH2 inhibition with tazemetostat in an animal model of melanoma reproduces the antimetastatic phenotype of AP-2α loss, suppressing anchorage-independent growth, reducing metastatic burden, and prolonging the animal survival, with durable effects in secondary xenografts. These findings define the AP-2α–E2F–EZH2 axis as a metastasis-specific transcriptional program and highlight AP-2α expression as a potential predictive biomarker for response to EZH2-target therapies, including in BRAF-wild-type and treatment-resistant melanoma [39]. Importantly, the therapeutic relevance of EZH2 inhibition is further supported by studies using ZLD1039, a potent and selective EZH2 inhibitor, which suppresses melanoma growth and lung metastasis by selectively reducing H3K27 methylation, inducing G0/G1 cell-cycle arrest via upregulation of p16 and p27, inhibiting CCND1/CDK6 and Cyclin E/CDK2 complexes, and promoting mitochondrial ROS–dependent apoptosis. ZLD1039 demonstrated robust antiproliferative and antimetastatic effects in both 2D and 3D models, as well as significant tumor suppression in A375 xenograft mice, accompanied by transcriptional reprogramming of cell-cycle, oxidative phosphorylation, and extracellular matrix interaction pathways, positioning EZH2 inhibition as a promising strategy to target both melanoma growth and metastatic progression [40].

### 3.7 Post-Translational Control of EZH2 Stability and Pigmentation-Linked Differentiation

Multiple regulatory layers converge to control EZH2 abundance and activity in melanoma, thereby shaping tumor phenotype, differentiation state, and therapeutic response. At the post-translational level, the UHRF1/UBE2L6/UBR4 axis emerges as a critical mechanism governing EZH2 stability. UHRF1-mediated promoter methylation suppresses *UBE2L6* expression in poorly pigmented melanoma cells, impairing UBE2L6–UBR4–dependent ubiquitination of EZH2 at K381 and preventing its proteasomal degradation. Restoration of UBE2L6 reduces EZH2 levels, promotes melanocytic differentiation, and markedly suppresses tumorigenicity and metastasis *in vivo*, with human melanoma samples showing an inverse correlation between UBE2L6 and EZH2 expression, supporting a tumor-suppressive role for this pathway [41].

### 3.8 Microenvironmental Stress: Hypoxia-Induced EZH2 Activation and Chemoresistance

In parallel, microenvironmental stress further reinforces EZH2-driven plasticity, as hypoxic conditions induce HIF-1α (Hypoxia-inducible factor 1α) expression, elevate EZH2 and H3K27me3 levels, and confer chemoresistance to SN-38 in murine melanoma cells. Genetic or pharmacologic inhibition of EZH2, including treatment with DZNep, suppresses hypoxia-induced H3K27me3 accumulation and restores chemosensitivity, indicating that EZH2-mediated epigenetic remodeling underlies adaptive resistance in hypoxic tumors. Collectively, these findings highlight EZH2 regulation through both post-translational control and microenvironment-induced activation as a central determinant of melanoma aggressiveness and therapy resistance and suggest that targeting EZH2 stability and function may be particularly effective in contexts where inhibition of its catalytic activity alone is insufficient [41].

### 3.9 NRAS-Mutant Melanoma and Bivalent Chromatin Dependencies

Molecular profiling studies indicate that NRAS is among the most common mutations in ALM, with a reported rate ranging from 10% to 30% of cases. These observations underscore the therapeutic potential of targeting epigenetic mechanisms, particularly in combined therapy, for the treatment of NRAS-mutant melanoma. Comprehensive epigenomic profiling, integrating 284 epigenomic maps with multi-omics analysis, revealed that NRAS-mutant melanomas are characterized by extensive bivalent chromatin marked by H3K27me3 and broad H3K4me3 domains. Reprogramming of bivalent H3K27me3 and its resolution through EZH2 inhibition using GSK-126, reduced invasive capacity and tumor burden. Although EZH2 inhibition produced only modest antitumor effects on invasion, its combination with MEK inhibition (trametinib) significantly decreased tumor burden in NRAS-mutant patient samples [42].

### 3.10 lncRNA_EZH2 Recruitment and Locus-Specific Epigenetic Silence Networks

In addition, there are indications that nuclear lncRNAs (long noncoding RNAs) and EZH2 form a central epigenetic silencing axis in melanoma, driving proliferation, invasion, metastasis, phenotypic plasticity, immune evasion, and therapeutic resistance. EZH2 is frequently amplified, overexpressed, or mutated in melanoma, promoting H3K27 trimethylation and repression of tumor suppressor genes, including *CDKN2A*, *CDKN2B* (cyclin-dependent kinase inhibitor 2B), *CDKN1A* (cyclin-dependent kinase inhibitor 1A), *PDCD4* (programmed cell death protein 4), CADM1 (cell adhesion molecule 1), and *RUNX3* (RUNX family transcription factor 3), while also modulating immune-response pathways and fostering T-cell exhaustion. In this context, multiple lncRNAs act as molecular “address codes” that recruit EZH2 to specific genomic loci: oncogenic lncRNAs such as *FOXC2-AS1* (FOXC2 antisense RNA 1), *CASC15* (cancer susceptibility candidate 15), *FALEC* (focally amplified long non-coding RNA in epithelial cancer), *HEIH* (high expression in hepatocellular carcinoma), *ILF3-AS1* (ILF3 antisense RNA 1), *PVT1* (plasmacytoma variant translocation 1), *MIR31HG* (MIR31 host gene), *LNMAT1* (lymph node metastasis associated transcript 1), and *HOXD-AS1* (HOXD antisense growth-associated long non-coding RNA 1) are upregulated in melanoma and promote silencing of tumor suppressor programs, whereas loss of tumor-suppressive lncRNAs such as *LINC-PINT* (long intergenic non-protein coding RNA, p53 induced transcript) and *GAS5* (growth arrest-specific transcript 5) abrogates PRC2-dependent repression of oncogenic pathways [43]. Through these interactions, the lncRNA–EZH2 network orchestrates cell-cycle progression, EMT-like transitions, migration, invasion, and immune escape. Importantly, given the limitations of global EZH2 inhibition, including off-target effects, EZH1 compensation, and noncanonical EZH2 functions, emerging therapeutic strategies aim to selectively disrupt lncRNA–EZH2 interactions, representing a promising paradigm for precision epigenetic therapy in melanoma [43].

### 3.11 Clinical Relevance and Emerging Evidence Supporting EZH2 Targeting in Melanoma

EZH2, the catalytic subunit of the PRC2, mediates trimethylation of histone H3 at lysine 27 (H3K27me3), promoting chromatin compaction and stable transcriptional repression, and in melanoma is frequently overexpressed or harbors gain-of-function alterations in up to 58% of tumors, contributing to tumor initiation, progression, and metastatic dissemination; although EZH2-targeted therapies are clinically established in follicular lymphoma and epithelioid sarcoma, their use in melanoma remains limited, with emerging clinical evidence, including a pediatric case harboring a missense A692V EZH2 mutation treated with tazemetostat as adjuvant, achieved prolonged recurrence-free survival, supporting EZH2 as a promising therapeutic target in this disease [44,45].

**Figure 2:**
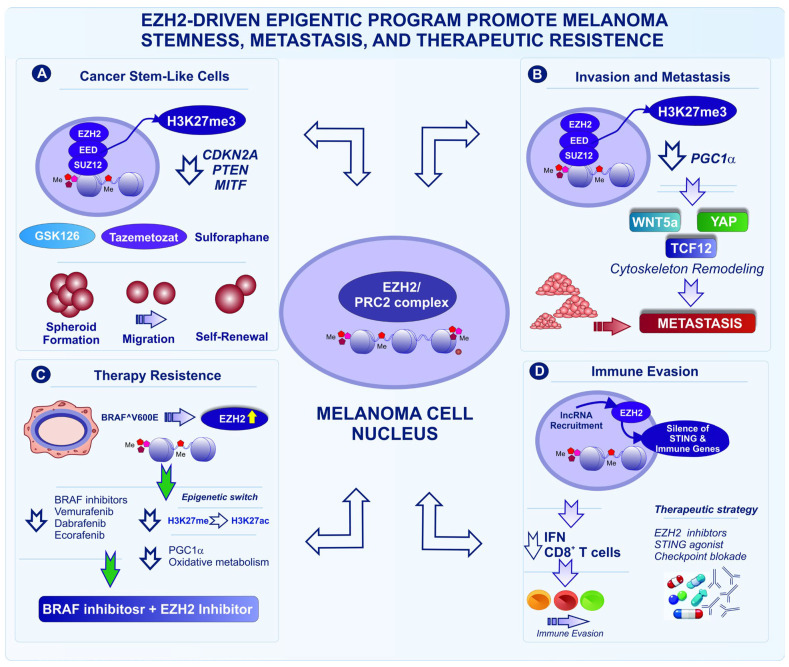
EZH2-driven epigenetic programs promote melanoma stemness, metastasis, therapeutic resistance, and immune evasion. (**A**) EZH2 sustains melanoma cancer stem-like cells by catalyzing H3K27 trimethylation (H3K27me3), repressing tumor suppressor and differentiation genes such as *CDKN2A*, *PTEN*, and *MITF* (*Microphthalmia-Associated Transcription Factor*), thereby promoting spheroid formation, migration, invasion, and self-renewal. Pharmacologic inhibition of EZH2 such as GSK126, tazemetostat or sulforaphane reduces stem-like properties, (**B**) EZH2-mediated H3K27me3 silencing of *PGC1α* (*Peroxisome Proliferator–Activated Receptor Gamma Coactivator 1-alpha*) activates invasive signaling pathways including WNT5A (Wnt Family Member 5A), YAP (Yes-Associated Protein), and TCF12 (Transcription Factor 12), promoting cytoskeletal remodeling and metastasis, (**C**) In BRAF^V600E melanoma, oncogenic signaling increases EZH2 expression and H3K27me3 accumulation, while targeted therapy induces epigenetic remodeling such as H3K27me3→H3K27ac and metabolic adaptation through the PGC1α axis, contributing to drug tolerance; combined BRAF and EZH2 inhibition represents a potential therapeutic strategy, (**D**) EZH2 also contributes to immune evasion by repressing STING (Stimulator of Interferon Genes)-dependent innate immune signaling and interferon responses, limiting CD8^+^ T-cell infiltration. Recruitment of EZH2 by oncogenic lncRNAs further promotes tumor suppressor gene silencing; EZH2 inhibition combined with STING agonist may restore immune activation and enhance responses to immunotherapy. Abb: EED (Embryonic Ectoderm Development), SUZ12 (Suppressor of Zeste 12 Protein Homolog), GSK126 (EZH2 methyltransferase inhibitor developed by GlaxoSmithKline), BRAF (B-Raf Proto-Oncogene, Serine/Threonine Kinase), IFN (Interferon).

Collectively, the evidence presented in these sections establishes EZH2-centered epigenetic regulation as a unifying mechanism that integrates cancer stemness, metabolic adaptation, metastatic competence, microenvironmental stress responses, and therapeutic resistance in melanoma (Fig. 2). Although many of these insights originate from cutaneous melanoma models, they are particularly consequential for ALM, a subtype characterized by low mutational burden, frequent structural alterations, and a relative paucity of actionable driver mutations. In ALM, where oncogenic signaling alone is insufficient to explain tumor aggressiveness and immune escape, the epigenetic plasticity driven by EZH2, its upstream regulators, and its interaction with transcription factors, noncoding RNAs, and metabolic programs, likely compensates for limited genetic diversity and could shape the disease progression. The convergence of EZH2-dependent chromatin remodeling with NRAS signaling, mechanotransduction, hypoxia, and immune modulation underscores epigenetic vulnerability as a central dependency of ALM. Importantly, these findings position epigenetic biomarkers and combinatorial epigenetic therapies, notably those targeting EZH2 activity, stability, or locus-specific recruitment, as particularly promising strategies to overcome intrinsic resistance and improve outcomes in this aggressive and therapeutically challenging melanoma subtype.

## 4 Relevance of EZH2 in Immunological Events in the Tumor Environment

This section aims to summarize how EZH2-dependent epigenetic regulation shapes immune dynamics within the melanoma TME. Specifically, it highlights how EZH2 integrates hypoxia signaling, innate immune sensing, antigen presentation, and T-cell functionality, thereby influencing immune exclusion, responsiveness to immunotherapy, and resistance to treatment. Understanding these mechanisms provides a framework for therapeutic strategies that combine EZH2 inhibition with immunomodulatory approaches to enhance anti-tumor immunity.

### 4.1 EZH2_HIF-1α Interaction and Macrophage Polarization in the TME

Targeting epigenetic-driven hypoxia and immune suppression has emerged as a powerful strategy to reprogram the melanoma TME and enhance anti-tumor immunity, HIF-1α and EZH2 have been shown to interact functionally in cancer, with HIF-1α modulating PRC2/EZH2 activity and EZH2 influencing HIF-1α expression and function, suggesting a regulatory feedback circuit that integrates hypoxia signaling with chromatin remodeling and gene repression in the TME [44]. Using *in vitro* assays, macrophage polarization analyses, and *in vivo* B16-F10 melanoma mouse models, the CRISPR/dCas9-EZH2 system-mediated repression of HIF1α in macrophages, promoted their reprogramming toward an M1-like, tumor-suppressive phenotype, enhancing phagocytic activity, reducing angiogenic factor expression, and reshaping the immune landscape by enhancing CD8^+^ T-cell activation while reducing PD-1 expression and regulatory T-cell. Collectively, HIF1α repression in macrophages significantly inhibited tumor growth, suppressed angiogenesis, and improved overall survival, demonstrating a potent strategy for enhancing cancer immunotherapy [46].

### 4.2 Epigenetic Repression of STING and Immune Exclusion in “Cold” Melanoma

In parallel, epigenetic silence mechanisms have been identified as central drivers of immune exclusion in so-called “*cold*” melanomas, which are characterized by poor T-cell infiltration, impaired antigen presentation, and attenuated type I interferon signaling. A key component of this process is the epigenetic repression of the innate immune sensor STING, frequently silenced in melanoma cells despite the absence of structural gene alterations. EZH2-mediated deposition of H3K27me3 at the STING locus limits activation of the TBK1–IRF3 pathway and downstream interferon responses. Pharmacologic inhibition of EZH2 restores STING expression, enhances type I interferon production, increases MHC-I expression and inflammatory chemokine secretion, and promotes CD8^+^ T-cell infiltration, effects that are further amplified when combined with STING agonists, effectively converting immunologically “cold” tumors into “hot” lesions with improved tumor control and survival in preclinical models [38,47].

### 4.3 PRC2-Mediated Repression of Antigen Presentation Pathways

Beyond innate immune sensing, PRC2-dependent epigenetic repression also directly impairs antigen presentation pathways. EZH2 and its cofactor JARID2 suppress MHC class II expression in melanoma cells by blocking the induction of CIITA, the master regulator of this pathway. Transcriptomic analyses of TCGA cohorts and metastatic melanomas treated with anti–PD-1 therapy revealed that tumors with high PRC2 activity exhibit reduced expression of antigen-presentation genes, diminished T-cell infiltration, and poor immunotherapy responsiveness. Mechanistically, EZH2 inhibition using agents such as tazemetostat opens chromatin at CIITA regulatory regions, enabling IFN-γ–driven MHC-II induction and restoring tumor immunogenicity. Clinically, elevated JARID2 expression correlates with low HLA-DR levels and inferior responses to PD-1 blockade, identifying PRC2 activity as a determinant of intrinsic resistance to immune checkpoint therapy [48].

### 4.4 Regulation of Interferon Responsiveness through IFI16

The epigenetic control of interferon responsiveness is further reinforced by regulation of IFI16, a cytosolic DNA sensor essential for maintaining STING expression and coordinating activation of the STING–TBK1–IRF3 and NF-κB pathways. EZH2-mediated H3K27me3 represses IFI16 in melanoma cells, whereas EZH2 inhibition removes this repression, priming tumor cells for robust IFN-γ responses marked by increased IFI16, STING, CXCL10, and ICAM1 expression. Additionally, high IFI16 expression in metastatic melanoma correlates with enhanced tumor inflammation, increased M1 macrophage infiltration, improved prognosis, and superior responses to anti–PD-1 therapy, positioning IFI16 as both a prognostic and predictive biomarker and supporting combination strategies incorporating EZH2 inhibitors to overcome primary immunotherapy resistance [49].

### 4.5 Mi-2β (CHD4)_EZH2 Axis and Interferon-Regulated Immune Evasion

Additional layers of epigenetic immune evasion are mediated by the NuRD complex component Mi-2β (CHD4), which represses interferon-stimulated genes and limits effector T-cell recruitment. Loss of Mi-2β increases CD8^+^ T-cell infiltration and sensitizes melanoma to PD-1 blockade, while pharmacologic inhibition using the Mi-2β ATPase inhibitor Z36-MP5 restores interferon-driven chemokine expression and synergizes with anti–PD-1 therapy. Notably, Mi-2β physically interacts with EZH2 and promotes its methylation, reinforcing H3K27me3-mediated silencing and underscoring the functional importance of the Mi-2β–EZH2 axis in checkpoint resistance [50].

Consistent with these observations, EZH2 inhibition has also been shown to reverse resistance to the chemotherapeutic and immune checkpoint therapies by modulating H3K27 methylation. Pharmacologic demethylation of H3K27 enhances sensitivity to paclitaxel and improves the efficacy of anti-TIGIT therapy by downregulating their ligand CD155, further highlighting the broad therapeutic impact of epigenetic reprogramming in melanoma [51].

### 4.6 Context-Dependent Effects of EZH2 Modulation on T-Cell Functionality

Tazemetostat induces dose- and time-dependent apoptosis in melanoma cells, reduces PRC2 complex components (EZH2, EED, SUZ12), and decreases H3K27me3 levels, with combinatorial regimens producing superior antitumor effects while sparing nonmalignant cells [52]. Importantly, EZH2 modulation also exerts context-dependent effects on T-cell biology. While genetic deletion of EZH2 impairs CD8^+^ T-cell expansion and effector function, transient pharmacologic inhibition of EZH2 prior to the onset of exhaustion preserves T-cell stemness, polyfunctionality, and responsiveness to PD-1 blockade. In murine melanoma models and human CAR-T systems, short-term exposure to tazemetostat reduced H3K27me3, increased TCF1 expression, delayed exhaustion, and significantly enhanced antitumor immunity without compromising proliferation, highlighting transient epigenetic reprogramming as a promising strategy to optimize immunotherapy outcomes [53].

Collectively, these findings establish EZH2 as a central epigenetic integrator of hypoxia signaling, innate immune sensing, antigen presentation, and T-cell functionality within the melanoma TME. By coordinating PRC2-dependent chromatin repression across multiple immune-regulatory pathways, including STING_IFI16_interferon signaling, MHC class I and II antigen presentation, macrophage polarization, and T-cell exhaustion, EZH2 actively shapes immune exclusion, therapeutic resistance, and tumor persistence (graphical abstract). Importantly, the context-dependent and reversible nature of EZH2-mediated epigenetic programs provides a unique therapeutic window: transient or combinatorial EZH2 inhibition can simultaneously reprogram tumor cells and immune compartments, converting immunologically “cold” melanomas into “hot”, treatment-responsive lesions. These insights position EZH2 not only as a mechanistic driver of immune escape but also as a strategic target for rational combination therapies designed to enhance the durability and efficacy of immunotherapy in melanoma.

## 5 Innovative Delivery and Microenvironment-Modulating Approaches

This section aims to discuss emerging strategies designed to enhance the therapeutic efficacy of EZH2 inhibition through advanced drug-delivery technologies and TME–modulating approaches. Emphasis is placed on nanotechnology-based systems, stimuli-responsive platforms, and cell-based delivery strategies that improve tumor specificity, optimize pharmacokinetics, and promote local immune reprogramming. These approaches aim to overcome the intrinsic limitations of systemic epigenetic therapy and to maximize the synergistic potential between EZH2 inhibitors, immunotherapies, and TME–targeting agents.

### 5.1 Limitations of Systemic EZH2 Inhibition and the Rationale for Targeted Delivery

Epigenetic therapy with EZH2 inhibitors, either as monotherapy or in combination with immune checkpoint blockade and TME-targeting immunomodulatory agents, holds significant therapeutic promise. However, these combinations have important challenges to face for translational therapy. These may include limited tumor specificity, systemic toxicity, suboptimal pharmacokinetics, and the immunosuppressive nature of the melanoma microenvironment, which collectively constrain the optimal therapeutic efficacy. These limitations may be overcome through innovative drug-delivery strategies designed to enhance tumor targeting, improve local drug concentration, and simultaneously reshape the TME.

### 5.2 Nanotechnology-Based and Stimuli-Responsive Delivery Platforms

Nanotechnology-based platforms, such as lipid nanoparticles, polymeric nanoparticles, micelles, and biomimetic carriers, enable controlled and sustained release of EZH2 inhibitors while reducing off-target exposure [54,55,56]. Functionalization of these carriers with tumor-homing ligands (e.g., integrin-binding peptides, chemokine receptor ligands, or antibodies against melanoma-associated antigens) further enhances selective accumulation within tumor tissues and metastatic niches [57,58,59].

In parallel, stimuli-responsive delivery systems that exploit intrinsic tumor characteristics, including acidic pH, hypoxia, elevated reactive oxygen species (ROS), or matrix metalloproteinase activity, allow on-demand drug release specifically within the TME [60,61,62,63]. Such approaches maximize local epigenetic reprogramming while minimizing systemic adverse effects.

### 5.3 Combinatorial Delivery Systems for TME Reprogramming

Beyond delivery optimization, combinatorial platforms integrating EZH2 inhibition with TME-modulating agents represent a particularly attractive strategy. Co-encapsulation of EZH2 inhibitors with immune checkpoint inhibitors, cytokines, STING agonists, or chemokine modulators can synergistically promote antigen presentation, enhance T-cell infiltration, and reverse immune exhaustion. Additionally, targeted delivery to stromal or myeloid compartments may reprogram tumor-associated macrophages and suppressive myeloid populations, thereby shifting the TME toward a pro-inflammatory and immunostimulatory state [64,65,66,67,68].

### 5.4 Cell-Based Delivery Systems and Integration with Adoptive Cellular Therapies

Emerging approaches such as cell-based delivery systems, including engineered T cells, macrophages, and extracellular vesicles, offer additional opportunities for precise spatial control of EZH2 inhibitor release while simultaneously providing active immunological engagement. Notably, in hematological malignancies, PRC2 inhibition in CAR-T cells has been shown to enhance therapeutic efficacy by promoting CD8^+^ T-cell function and persistence [69], highlighting the translational potential of integrating epigenetic modulation directly within adoptive cellular therapies. These platforms are particularly appealing in melanoma, where adoptive cell therapies and chemokine-guided trafficking can be leveraged to deliver epigenetic modulators directly to tumor sites.

Collectively, the integration of advanced drug-delivery technologies with microenvironment-reprogramming strategies has the potential to substantially enhance the therapeutic index of EZH2 inhibition and its combinations with immunotherapy. Such multimodal approaches may ultimately overcome resistance mechanisms, improve T-cell functionality and persistence, and enable more durable clinical responses in melanoma and other epigenetically driven malignancies.

## 6 EZH2 Current Clinical Trials

Recent advances in epigenetic therapy have positioned EZH2 inhibitors as promising agents for melanoma treatment. Tazemetostat, the first-in-class EZH2 inhibitor, has already been approved for follicular lymphoma and epithelioid sarcoma, and is currently being evaluated in a phase II trial for melanoma. As monotherapy, tazemetostat reduces repression of tumor suppressor genes and restores the expression of tumor-associated antigens, thereby counteracting one of the key mechanisms of immune evasion in melanoma (Table 2).

**Table 2:** Enhancer of Zeste Homolog 2 (EZH2) inhibitors in clinical trials.

Enzyme	Inhibitor	Combination	Clinical Phase	Potential Benefit
EZH2	Tazemetostat	None	Approved for lymphoma; Phase II in melanoma	Reduces repression of tumor suppressor genes, restores tumor antigens
EZH2	Tazemetostat	Anti-PD-1 (Embrolizumab)	Phase II (NCT04705818)	Reactivates tumor antigens, increases MHC-I/II expression
EZH2	CPI-1205	Ipilimumabe (anti-CTLA-4)	Phase I/II (NCT03525795)	Reduces Tregs intratumorally, enhances T-cell activation

**Abb:** PD-1 (Programmed cell death protein 1); CTLA-4 (cytotoxic T-lymphocyte-associated protein 4); MHC (Major Histocompatibility Complex).

Beyond monotherapy, combinatorial approaches have shown better responses. Tazemetostat is being investigated in combination with anti-PD-1 therapy (Pembrolizumab) in a phase II trial. This strategy aims to enhance immunogenicity by reactivating tumor antigens and increasing MHC-I and MHC-II expression, thus improving the efficacy of checkpoint blockade in tumors with low baseline immunogenicity (Table 2). Similarly, the EZH2 inhibitor CPI-1205 is under evaluation in combination with Ipilimumab (anti-CTLA-4) in phase I/II studies. Preclinical evidence suggests that this combination reduces the frequency of intratumoral regulatory T cells and enhances effector T-cell activation, potentially overcoming resistance to checkpoint inhibitors (Table 2).

Together, these early clinical trials underscore the therapeutic relevance of EZH2 inhibition in melanoma. While single-agent activity appears modest, the integration of EZH2 inhibitors into immunotherapy-based regimens holds significant promise to enhance antitumor responses, particularly in challenging subtypes such as acral and mucosal melanoma.

With respect to safety, tazemetostat has demonstrated a generally manageable toxicity profile in both approved indications and early-phase (I/II) trials. Grade ≥ 3 treatment-related adverse events (TRAEs) occur at variable rates but typically low frequencies < 20% in lymphoma/sarcoma, and serious events are rare but reported, including anemia, weight loss, and rare neurological toxicities. When combined with immune checkpoint inhibitors, the incidence of immune-mediated toxicities increases, underscoring the need for careful monitoring [70].

## 7 Challenges and Future Perspectives of EZH2-Targeted Therapies in ALM

### 7.1 Challenges

Despite the compelling preclinical rationale supporting EZH2 as a therapeutic target in melanoma, several limitations must be critically considered when translating EZH2-directed strategies to ALM. A major challenge is the marked heterogeneity in EZH2 dependency and therapeutic response, which likely reflects the copy-number–driven, structurally complex genomic landscape of ALM rather than uniform oncogene addiction [71,72,73]. Unlike cutaneous melanoma, where specific driver mutations can guide targeted therapy, ALM exhibits variable EZH2 amplification, expression, and chromatin dependency, complicating patient selection and response prediction.

Another important limitation is the paucity of ALM-specific clinical data. Most available evidence supporting EZH2 inhibition derives from cutaneous melanoma models or other malignancies, with ALM patients underrepresented or absent from clinical trials evaluating EZH2 inhibitors. Consequently, the magnitude and durability of clinical benefit in ALM remain uncertain, underscoring the need for ALM-enriched cohorts, biomarker-driven trial designs, and co-clinical studies incorporating biologically faithful ALM models.

Emerging data also suggest that adaptive and intrinsic resistance mechanisms may limit the efficacy of EZH2-targeted monotherapy. These include compensatory activity of EZH1, activation of alternative repressive complexes, and engagement of noncanonical EZH2 functions independent of PRC2-mediated H3K27 trimethylation. In addition, prolonged EZH2 inhibition may induce chromatin reprogramming that favors cellular plasticity, allowing tumor cells to adopt alternative transcriptional states rather than undergoing sustained growth arrest or immune elimination. These observations argue strongly against EZH2 inhibition as a standalone strategy in ALM and instead support its use within rational combination regimens.

From a mechanistic perspective, EZH2 does not operate in isolation but is embedded within a broader epigenetic network that includes DNA methyltransferases (DNMTs), histone demethylases, and chromatin remodeling complexes [74,75,76]. In ALM, where promoter hypermethylation and widespread copy-number alterations are prevalent, EZH2-mediated repression frequently cooperates with DNMT-driven DNA methylation, reinforcing stable silencing of tumor suppressor and immune-related genes. Similarly, functional interplay between EZH2 and histone demethylases such as KDM6A/UTX or KDM6B/JMJD3, demonstrated in several cancer types, can dynamically regulate H3K27 methylation states, influencing transcriptional plasticity, metabolic adaptation, and immune responsiveness [77]. Disruption of this balance may determine whether EZH2 inhibition leads to durable reprogramming or transient chromatin relaxation followed by relapse.

Finally, EZH2 intersects with signaling pathways particularly relevant to ALM, including NRAS–MAPK signaling, mechanotransduction-associated pathways, and stress-response programs activated in anatomically constrained acral sites. These interactions may shape context-specific dependencies on EZH2 activity and influence therapeutic vulnerability. A deeper understanding of how EZH2 integrates with these pathways will be essential to define optimal combination strategies and avoid ineffective or antagonistic therapeutic pairings.

In summary, while EZH2 represents a promising epigenetic target in ALM, its clinical translation faces significant challenges related to biological heterogeneity, resistance mechanisms, and limited subtype-specific data. Addressing these limitations will require integrative approaches combining epigenetic, genomic, and immunologic profiling, alongside ALM-focused experimental models and carefully designed clinical studies. Such efforts will be critical to determine whether EZH2 inhibition can be effectively leveraged as part of precision, combination-based therapies for this aggressive and underserved melanoma subtype.

### 7.2 Future Perspectives

Emerging insights into EZH2-driven epigenetic plasticity have opened a promising therapeutic landscape for ALM, a subtype historically limited by low mutational burden and restricted treatment options. Accumulating preclinical and translational evidence supports the concept that rational combination strategies integrating EZH2 inhibition with immune-based therapies such as anti–PD-1 or anti–CTLA-4 antibodies, STING agonists, or other innate immune activators have strong potential to convert epigenetically silenced, immune-excluded ALM tumors into immunologically responsive disease. In this context, EZH2 inhibition offers a unique mechanism to restore antigen presentation, interferon signaling, and cytotoxic T-cell infiltration, thereby enhancing both the depth and durability of immunotherapeutic responses.

Importantly, the expanding molecular understanding of EZH2 regulation in melanoma enables a biomarker-informed path to clinical translation in ALM. Early-phase clinical studies can be designed with integrated pharmacodynamic endpoints, such as reductions in H3K27me3 levels, induction of interferon-stimulated gene programs, and increased CD8^+^ T-cell infiltration to directly link epigenetic reprogramming with immune activation. Stratification based on EZH2 copy-number gain, global H3K27me3 burden, transcriptional repression signatures, or immune-exclusion phenotypes may be further enriched for patients most likely to benefit, increasing the probability of demonstrating meaningful clinical synergy.

Progress in this field is further accelerated by advances in experimental modeling. The development of immunocompetent allograft systems, patient-derived organoids, and *ex vivo* ALM explant cultures provides biologically faithful platforms to interrogate tumor–immune interactions, validate biomarkers, and optimize therapeutic sequencing in a co-clinical framework.

In parallel, standardization of EZH2- and H3K27me3-based analytical assays across centers will facilitate reproducibility and regulatory readiness. Collectively, the convergence of mechanistic insight, biomarker-driven trial design, and next-generation model systems positions EZH2-targeted strategies as a realistic and transformative avenue for improving outcomes in ALM, shifting the paradigm from therapeutic scarcity toward precision epigenetic immunotherapy.

## 8 Conclusion

ALM exhibits a unique molecular profile, characterized by a low mutational burden, structural chromosomal alterations, and a strong reliance on epigenetic mechanisms. In this context, EZH2 emerges as a central target, modulating both gene expression and tumor–immune system interactions. Despite progress in the development of epigenetic inhibitors, clinical outcomes in melanoma remain preliminary and limited. The modest efficacy of EZH2 inhibitors as monotherapy reinforces the rationale for their use in combination with immunotherapy. Moreover, the design of clinical trials specifically dedicated to ALM is critical, given the poor response of this subtype to current treatments. Key gaps include the identification of predictive biomarkers of response, the assessment of synergy between epigenetic modulation and immunotherapy, and the development of representative preclinical models of ALM. The repositioning of epigenetic inhibitors in combination with immunotherapies represents one of the most promising strategies to overcome therapeutic resistance in ALM.

## Data Availability

Not applicable.

## References

[1] Bray F , Laversanne M , Sung H , Ferlay J , Siegel RL , Soerjomataram I , et al. Global cancer statistics 2022: GLOBOCAN estimates of incidence and mortality worldwide for 36 cancers in 185 countries. CA Cancer J Clin. 2024; 74( 3): 229– 63. doi:10.3322/caac.21834. 38572751

[2] Caraviello C , Nazzaro G , Tavoletti G , Boggio F , Denaro N , Murgia G , et al. Melanoma skin cancer: A comprehensive review of current knowledge. Cancers. 2025; 17( 17): 2920. doi:10.3390/cancers17172920. 40941017 PMC12427887

[3] Qin Z , Zheng M . Advances in targeted therapy and immunotherapy for melanoma (Review). Exp Ther Med. 2023; 26( 3): 416. doi:10.3892/etm.2023.12115. 37559935 PMC10407994

[4] Li C , Lin X , Wang J , Zhou Q , Feng F , Xu J . Multi-omics-based subtyping of melanoma suggests distinct immune and targeted therapy strategies. Front Immunol. 2025; 16: 1601243. doi:10.3389/fimmu.2025.1601243. 40574832 PMC12197953

[5] Udayakumar D , Tsao H . Melanoma genetics: An update on risk-associated genes. Hematol Oncol Clin North Am. 2009; 23( 3): 415– 29. doi:10.1016/j.hoc.2009.03.011. 19464594

[6] Guo Y , Chen Y , Zhang L , Ma L , Jiang K , Yao G , et al. TERT promoter mutations and telomerase in melanoma. J Oncol. 2022; 2022: 6300329. doi:10.1155/2022/6300329. 35903534 PMC9325578

[7] Liang WS , Hendricks W , Kiefer J , Schmidt J , Sekar S , Carpten J , et al. Integrated genomic analyses reveal frequent TERT aberrations in acral melanoma. Genome Res. 2017; 27( 4): 524– 32. doi:10.1101/gr.213348.116. 28373299 PMC5378171

[8] Wang M , Fukushima S , Sheen YS , Ramelyte E , Cruz-Pacheco N , Shi C , et al. The genetic evolution of acral melanoma. Nat Commun. 2024; 15: 6146. doi:10.1038/s41467-024-50233-z. 39034322 PMC11271482

[9] Park HS , Kim JH , Cho MY , Chung KY , Roh MR . PTEN promoter hypermethylation is associated with Breslow thickness in acral melanoma on the heel, forefoot, and hallux. Ann Dermatol. 2021; 33( 1): 18– 25. doi:10.5021/ad.2021.33.1.18. 33911808 PMC7875221

[10] Fariyike O , Johnson NA , Craig P , Nobes J , Levell NJ , Venables Z . Understanding acral lentiginous melanoma: From clinic to guidelines. Clin Exp Dermatol. 2025; llaf501. doi:10.1093/ced/llaf501. 41219165

[11] Margueron R , Reinberg D . The Polycomb complex PRC2 and its mark in life. Nature. 2011; 469( 7330): 343– 9. doi:10.1038/nature09784. 21248841 PMC3760771

[12] Tiffen J , Gallagher SJ , Hersey P . EZH2: An emerging role in melanoma biology and strategies for targeted therapy. Pigment Cell Melanoma Res. 2015; 28( 1): 21– 30. doi:10.1111/pcmr.12280. 24912396

[13] Tiffen JC , Gunatilake D , Gallagher SJ , Gowrishankar K , Heinemann A , Cullinane C , et al. Targeting activating mutations of EZH2 leads to potent cell growth inhibition in human melanoma by derepression of tumor suppressor genes. Oncotarget. 2015; 6( 29): 27023– 36. doi:10.18632/oncotarget.4809. 26304929 PMC4694971

[14] Broit N , Johansson PA , Rodgers CB , Walpole ST , Hayward NK , Pritchard AL . Systematic review and meta-analysis of genomic alterations in acral melanoma. Pigment Cell Melanoma Res. 2022; 35( 3): 369– 86. doi:10.1111/pcmr.13034. 35229492 PMC9540316

[15] Bollu VS , Chen YC , Zhang F , Gowda K , Amin S , Sharma AK , et al. Managing telomerase and telomere dysfunction in acral melanoma. Pharmacol Res. 2025; 215: 107700. doi:10.1016/j.phrs.2025.107700. 40097124

[16] Kim SH , Tsao H . Acral melanoma: A review of its pathogenesis, progression, and management. Biomolecules. 2025; 15( 1): 120. doi:10.3390/biom15010120. 39858514 PMC11763010

[17] Liu H , Gao J , Feng M , Cheng J , Tang Y , Cao Q , et al. Integrative molecular and spatial analysis reveals evolutionary dynamics and tumor-immune interplay of *in situ* and invasive acral melanoma. Cancer Cell. 2024; 42( 6): 1067– 85.e11. doi:10.1016/j.ccell.2024.04.012. 38759655

[18] Zingg D , Arenas-Ramirez N , Sahin D , Rosalia RA , Antunes AT , Haeusel J , et al. The histone methyltransferase Ezh2 controls mechanisms of adaptive resistance to tumor immunotherapy. Cell Rep. 2017; 20( 4): 854– 67. doi:10.1016/j.celrep.2017.07.007. 28746871

[19] Flesher JL , Fisher DE . G9a: An emerging epigenetic target for melanoma therapy. Epigenomes. 2021; 5( 4): 23. doi:10.3390/epigenomes5040023. 34691767 PMC8536146

[20] Cheng PF . Medical bioinformatics in melanoma. Curr Opin Oncol. 2018; 30( 2): 113– 7. doi:10.1097/CCO.0000000000000428. 29227308

[21] Pradhan D , Jour G , Milton D , Vasudevaraja V , Tetzlaff MT , Nagarajan P , et al. Aberrant DNA methylation predicts melanoma-specific survival in patients with acral melanoma. Cancers. 2019; 11( 12): 2031. doi:10.3390/cancers11122031. 31888295 PMC6966546

[22] Tigu AB , Ivancuta A , Uhl A , Sabo AC , Nistor M , Mureșan XM , et al. Epigenetic therapies in melanoma—Targeting DNA methylation and histone modification. Biomedicines. 2025; 13( 5): 1188. doi:10.3390/biomedicines13051188. 40427015 PMC12108579

[23] McCabe MT , Ott HM , Ganji G , Korenchuk S , Thompson C , Van Aller GS , et al. EZH2 inhibition as a therapeutic strategy for lymphoma with EZH2-activating mutations. Nature. 2012; 492( 7427): 108– 12. doi:10.1038/nature11606. 23051747

[24] Shi X , Tasdogan A , Huang F , Hu Z , Morrison SJ , DeBerardinis RJ . The abundance of metabolites related to protein methylation correlates with the metastatic capacity of human melanoma xenografts. Sci Adv. 2017; 3( 11): eaao5268. doi:10.1126/sciadv.aao5268. 29109980 PMC5665593

[25] Mahmoud F , Shields B , Makhoul I , Hutchins LF , Shalin SC , Tackett AJ . Role of EZH2 histone methyltrasferase in melanoma progression and metastasis. Cancer Biol Ther. 2016; 17( 6): 579– 91. doi:10.1080/15384047.2016.1167291. 27105109 PMC4990393

[26] Orouji E , Utikal J . Tackling malignant melanoma epigenetically: Histone lysine methylation. Clin Epigenetics. 2018; 10( 1): 145. doi:10.1186/s13148-018-0583-z. 30466474 PMC6249913

[27] Kooistra SM , Helin K . Molecular mechanisms and potential functions of histone demethylases. Nat Rev Mol Cell Biol. 2012; 13( 5): 297– 311. doi:10.1038/nrm3327. 22473470

[28] Tiffen JC , Gallagher SJ , Tseng HY , Filipp FV , Fazekas de St Groth B , Hersey P . EZH2 as a mediator of treatment resistance in melanoma. Pigment Cell Melanoma Res. 2016; 29( 5): 500– 7. doi:10.1111/pcmr.12481. 27063195 PMC5021620

[29] Kato S , Maeda Y , Sugiyama D , Watanabe K , Nishikawa H , Hinohara K . The cancer epigenome: Non-cell autonomous player in tumor immunity. Cancer Sci. 2023; 114( 3): 730– 40. doi:10.1111/cas.15681. 36468774 PMC9986067

[30] Fisher ML , Adhikary G , Grun D , Kaetzel DM , Eckert RL . The Ezh2 polycomb group protein drives an aggressive phenotype in melanoma cancer stem cells and is a target of diet derived sulforaphane. Mol Carcinog. 2016; 55( 12): 2024– 36. doi:10.1002/mc.22448. 26693692 PMC4919248

[31] Montagnani V , Benelli M , Apollo A , Pescucci C , Licastro D , Urso C , et al. Thin and thick primary cutaneous melanomas reveal distinct patterns of somatic copy number alterations. Oncotarget. 2016; 7( 21): 30365– 78. doi:10.18632/oncotarget.8758. 27095580 PMC5058686

[32] Luo C , Balsa E , Perry EA , Liang J , Tavares CD , Vazquez F , et al. H3K27me3-mediated PGC1α gene silencing promotes melanoma invasion through WNT5A and YAP. J Clin Investig. 2019; 130( 2): 853– 62. doi:10.1172/JCI130038. PMC6994149 31929186

[33] Hoffmann F , Fröhlich A , Schäfer N , Keil VC , Landsberg J , Herrlinger U , et al. Treatment of metastasized melanoma with combined checkpoint inhibition in a patient with highly active multiple sclerosis. J Dermatol. 2020; 47( 5): e184– 5. doi:10.1111/1346-8138.15272. 32096240

[34] Grigore F , Yang H , Hanson ND , VanBrocklin MW , Sarver AL , Robinson JP . BRAF inhibition in melanoma is associated with the dysregulation of histone methylation and histone methyltransferases. Neoplasia. 2020; 22( 9): 376– 89. doi:10.1016/j.neo.2020.06.006. 32629178 PMC7338995

[35] Uebel A , Kewitz-Hempel S , Willscher E , Gebhardt K , Sunderkötter C , Gerloff D . Resistance to BRAF inhibitors: EZH2 and its downstream targets as potential therapeutic options in melanoma. Int J Mol Sci. 2023; 24( 3): 1963. doi:10.3390/ijms24031963. 36768289 PMC9916477

[36] Zhou J , Chai X , Zhu Y , Huang Z , Lin T , Hu Z , et al. A methyl-to-acetyl switch in H3K27 drives metabolic reprogramming and resistance to BRAFV600E inhibition in melanoma. Neoplasia. 2025; 68: 101223. doi:10.1016/j.neo.2025.101223. 40850308 PMC12446969

[37] Song J , Yang P , Chen C , Ding W , Tillement O , Bai H , et al. Targeting epigenetic regulators as a promising avenue to overcome cancer therapy resistance. Sig Transduct Target Ther. 2025; 10: 219. doi:10.1038/s41392-025-02266-z. PMC12271501 40675967

[38] Al Emran A , Fisher DE . Dual targeting with EZH2 inhibitor and STING agonist to treat melanoma. J Investig Dermatol. 2022; 142( 4): 1004– 6. doi:10.1016/j.jid.2021.09.028. 35131084 PMC8957612

[39] White JR , Thompson DT , Koch KE , Kiriazov BS , Beck AC , van der Heide DM , et al. AP-2α–mediated activation of E2F and EZH2 drives melanoma metastasis. Cancer Res. 2021; 81( 17): 4455– 70. doi:10.1158/0008-5472.CAN-21-0772. 34210752 PMC8416798

[40] Xu T , Dai J , Tang L , Yang L , Si L , Sheng X , et al. EZH2 inhibitor enhances the STING agonist‒induced antitumor immunity in melanoma. J Investig Dermatol. 2022; 142( 4): 1158– 70.e8. doi:10.1016/j.jid.2021.08.437. 34571002

[41] Kuser-Abali G , Zhang Y , Szeto P , Zhao P , Masoumi-Moghaddam S , Fedele CG , et al. UHRF1/UBE2L6/UBR4-mediated ubiquitination regulates EZH2 abundance and thereby melanocytic differentiation phenotypes in melanoma. Oncogene. 2023; 42( 17): 1360– 73. doi:10.1038/s41388-023-02631-8. 36906655 PMC10121471

[42] Terranova CJ . Chromatin state profiling reveals PRC2 inhibition as a therapeutic target in NRAS-mutant melanoma. Mol Cell Oncol. 2021; 8( 5): 1986350. doi:10.1080/23723556.2021.1986350. 34859147 PMC8632323

[43] Wozniak M , Czyz M . lncRNAs-EZH2 interaction as promising therapeutic target in cutaneous melanoma. Front Mol Biosci. 2023; 10: 1170026. doi:10.3389/fmolb.2023.1170026. 37325482 PMC10265524

[44] Zingg D , Debbache J , Peña-Hernández R , Antunes AT , Schaefer SM , Cheng PF , et al. EZH2-mediated primary cilium deconstruction drives metastatic melanoma formation. Cancer Cell. 2018; 34( 1): 69– 84.e14. doi:10.1016/j.ccell.2018.06.001. 30008323

[45] Resch EE , Makri SC , Ghanem P , Baraban EG , Cohen KJ , Cohen AR , et al. Relapse-free survival in a pediatric patient with recurrent EZH2-mutant melanoma treated with adjuvant tazemetostat. npj Precis Onc. 2025; 9: 48. doi:10.1038/s41698-025-00826-8. PMC11845573 39984702

[46] Dong Y , Zhang S , Gao X , Yin D , Wang T , Li Z , et al. HIF1α epigenetically repressed macrophages via CRISPR/Cas9-EZH2 system for enhanced cancer immunotherapy. Bioact Mater. 2021; 6( 9): 2870– 80. doi:10.1016/j.bioactmat.2021.02.008. 33718668 PMC7905236

[47] Hosokawa M , Tetsumoto S , Yasui M , Kono Y , Ogawara KI . 3-deazaneplanocin A, a histone methyltransferase inhibitor, improved the chemoresistance induced under hypoxia in melanoma cells. Biochem Biophys Res Commun. 2023; 677: 26– 30. doi:10.1016/j.bbrc.2023.08.003. 37542772

[48] James JL , Taylor BC , Axelrod ML , Sun X , Guerin LN , Gonzalez-Ericsson PI , et al. Polycomb repressor complex 2 suppresses interferon-responsive MHC-II expression in melanoma cells and is associated with anti-PD-1 resistance. J Immunother Cancer. 2023; 11( 11): e007736. doi:10.1136/jitc-2023-007736. 38315170 PMC10660662

[49] Kobayashi Y , Bustos MA , Hayashi Y , Yu Q , Hoon D . Interferon-induced factor 16 is essential in metastatic melanoma to maintain STING levels and the immune responses upon IFN-γ response pathway activation. J Immunother Cancer. 2024; 12( 10): e009590. doi:10.1136/jitc-2024-009590. 39424359 PMC11492949

[50] Jia DD , Li T . Comprehensive insights on genetic alterations and immunotherapy prognosis in Chinese melanoma patients. Sci Rep. 2024; 14: 16607. doi:10.1038/s41598-024-65065-6. 39025927 PMC11258252

[51] Tang M , Duan T , Lu Y , Liu J , Gao C , Wang R . Tyrosinase-woven melanin nets for melanoma therapy through targeted mitochondrial tethering and enhanced photothermal treatment. Adv Mater. 2024; 36( 44): 2411906. doi:10.1002/adma.202411906. 39285827

[52] Anestopoulos I , Paraskevaidis I , Kyriakou S , Giova LE , Trafalis DT , Botaitis S , et al. Isothiocyanates potentiate tazemetostat-induced apoptosis by modulating the expression of apoptotic genes, members of polycomb repressive complex 2, and levels of tri-methylating lysine 27 at histone 3 in human malignant melanoma cells. Int J Mol Sci. 2024; 25( 5): 2745. doi:10.3390/ijms25052745. 38473991 PMC10931595

[53] Hou Y , Zak J , Shi Y , Pratumchai I , Dinner B , Wang W , et al. Transient EZH2 suppression by tazemetostat duringIn VitroExpansion maintains T-cell stemness and improves adoptive T-cell therapy. Cancer Immunol Res. 2025; 13( 1): 47– 65. doi:10.1158/2326-6066.CIR-24-0089. 39365901 PMC11717634

[54] Guo Y , Huang J , Lin M , Yin Q , Zhang T , Guo Z , et al. Nano particle loaded EZH2 inhibitors: Increased efficiency and reduced toxicity for malignant solid tumors. J Transl Int Med. 2025; 13( 2): 156– 69. doi:10.1515/jtim-2025-0020. 40443399 PMC12116265

[55] Milewska S , Sadowska A , Stefaniuk N , Misztalewska-Turkowicz I , Wilczewska AZ , Car H , et al. Tumor-homing peptides as crucial component of magnetic-based delivery systems: Recent developments and pharmacoeconomical perspective. Int J Mol Sci. 2024; 25( 11): 6219. doi:10.3390/ijms25116219. 38892406 PMC11172452

[56] Zhang J , Huang L , Ge G , Hu K . Emerging epigenetic-based nanotechnology for cancer therapy: Modulating the tumor microenvironment. Adv Sci. 2023; 10( 7): e2206169. doi:10.1002/advs.202206169. PMC9982594 36599655

[57] Gao T , Fu S , Quan X , Sun J , Jiang M , Li J . Advancing epigenetic combination therapy in oncology: Multifunctional nano-drug delivery systems for synergistic efficacy and precision modulation. Int J Nanomed. 2025; 20: 14853– 83. doi:10.2147/IJN.S566173. PMC12704357 41404378

[58] Rocchi A , Teesalu T , Celia C . Advancing cancer-targeted nanotherapies with tumor homing peptides. ACS Pharmacol Transl Sci. 2025; 8( 7): 1919– 33. doi:10.1021/acsptsci.5c00241. 40672665 PMC12261227

[59] Wang B , Tang D , Cui J , Jiang H , Yu J , Guo Z . RGD-based self-assembling nanodrugs for improved tumor therapy. Front Pharmacol. 2024; 15: 1477409. doi:10.3389/fphar.2024.1477409. 39411070 PMC11473307

[60] Zhang Y , Xing J , Jiang J , Liao M , Pan G , Wang Y . Hypoxia-responsive nanoparticles for fluorescence diagnosis and therapy of cancer. Theranostics. 2025; 15( 4): 1353– 75. doi:10.7150/thno.104190. 39816693 PMC11729551

[61] Liu Y , Si L , Jiang Y , Jiang S , Zhang X , Li S , et al. Design of pH-responsive nanomaterials based on the tumor microenvironment. Int J Nanomed. 2025; 20: 705– 21. doi:10.2147/IJN.S504629. PMC11752822 39845771

[62] Guo F , Du Y , Wang Y , Wang M , Wang L , Yu N , et al. Targeted drug delivery systems for matrix metalloproteinase-responsive anoparticles in tumor cells: A review. Int J Biol Macromol. 2024; 257( Pt 1): 128658. doi:10.1016/j.ijbiomac.2023.128658. 38065446

[63] Shi Y , Yu Q , Tan L , Wang Q , Zhu WH . Tumor microenvironment-responsive polymer delivery platforms for cancer therapy. Angew Chem Int Ed. 2025; 64( 26): e202503776. doi:10.1002/anie.202503776. 40214115

[64] Chen X , Xu Z , Li T , Thakur A , Wen Y , Zhang K , et al. Nanomaterial-encapsulated STING agonists for immune modulation in cancer therapy. Biomark Res. 2024; 12: 2. doi:10.1186/s40364-023-00551-z. 38185685 PMC10773049

[65] Nguyen NT , Le XT , Lee WT , Lim YT , Oh KT , Lee ES , et al. STING-activating dendritic cell-targeted nanovaccines that evoke potent antigen cross-presentation for cancer immunotherapy. Bioact Mater. 2024; 42: 345– 65. doi:10.1016/j.bioactmat.2024.09.002. 39290338 PMC11406000

[66] Zhao C , Pang X , Yang Z , Wang S , Deng H , Chen X . Nanomaterials targeting tumor associated macrophages for cancer immunotherapy. J Control Release. 2022; 341: 272– 84. doi:10.1016/j.jconrel.2021.11.028. 34813877

[67] Kuznetsova AB , Kolesova EP , Parodi A , Zamyatnin AA Jr , Egorova VS . Reprogramming tumor-associated macrophage using nanocarriers: New perspectives to halt cancer progression. Pharmaceutics. 2024; 16( 5): 636. doi:10.3390/pharmaceutics16050636. 38794298 PMC11124960

[68] Chen ZY , Liu HZ , Shang ZJ , Luo GF , Zhang XZ . Nanomedicine for targeting cancer-associated fibroblasts in cancer therapy. Theranostics. 2026; 16( 3): 1545– 76. doi:10.7150/thno.120283. 41355964 PMC12679702

[69] Rodrigues Carvalho ML , de Oliveira Andrade C , Cabral-Piccin MP , Kinker GS , Vitiello GAF , Gonçalves Cambuí RA , et al. Targeting PRC2 enhances the cytotoxic capacity of anti-CD19 CAR T cells against hematologic malignancies. Cancer Res. 2025; 85( 12): 2234– 49. doi:10.1158/0008-5472.CAN-24-1643. 39970330

[70] Gounder M , Schöffski P , Jones RL , Agulnik M , Cote GM , Villalobos VM , et al. Tazemetostat in advanced epithelioid sarcoma with loss of INI1/SMARCB1: An international, open-label, phase 2 basket study. Lancet Oncol. 2020; 21( 11): 1423– 32. doi:10.1016/S1470-2045(20)30451-4. 33035459

[71] Ng MF , Simmons JL , Boyle GM . Heterogeneity in melanoma. Cancers. 2022; 14( 12): 3030. doi:10.3390/cancers14123030. 35740696 PMC9221188

[72] Carvalho LAD , Aguiar FC , Smalley KSM , Possik PA . Acral melanoma: New insights into the immune and genomic landscape. Neoplasia. 2023; 46: 100947. doi:10.1016/j.neo.2023.100947. 37913653 PMC10637990

[73] Izsak A , Giles KM , Lui KP , Weiss SA , Moran U , Vega-Saenz de Miera E , et al. Targeting EZH2 in acral lentiginous melanoma (ALM). J Clin Oncol. 2017; 35( 15 Suppl): 9534. doi:10.1200/JCO.2017.35.15_suppl.9534.

[74] Tiffen J , Gallagher SJ , Filipp F , Gunatilake D , Al Emran A , Cullinane C , et al. EZH2 cooperates with DNA methylation to downregulate key tumor suppressors and IFN gene signatures in melanoma. J Investig Dermatol. 2020; 140( 12): 2442– 54.e5. doi:10.1016/j.jid.2020.02.042. 32360600

[75] Verma S , Goyal N , Goyal S , Kaur P , Gupta S . EZH2 dysregulation and its oncogenic role in human cancers. Cancers. 2025; 17( 19): 3111. doi:10.3390/cancers17193111. 41097640 PMC12523533

[76] Kang MK , Mehrazarin S , Park NH , Wang CY . Epigenetic gene regulation by histone demethylases: Emerging role in oncogenesis and inflammation. Histone demethylases in oncogenesis. Oral Dis. 2017; 23( 6): 709– 20. doi:10.1111/odi.12569. 27514027 PMC5493521

[77] Sherry-Lynes MM , Sengupta S , Kulkarni S , Cochran BH . Regulation of the JMJD3 (KDM6B) histone demethylase in glioblastoma stem cells by STAT3. PLoS One. 2017; 12( 4): e0174775. doi:10.1371/journal.pone.0174775. 28384648 PMC5383422

